# Sixth Year Molars

**Published:** 1866-04

**Authors:** J. H. Paine

**Affiliations:** Xenia, O.


### SIXTH YEAR MOLARS.

J. H. Paine, Xenia, O.

The First Permanent Molars, commonly called the "Sixth Year Molars," is assigned as a subject for essay upon this occasion."*

These are four in number, two in each Maxillary; are usually erupted between the fifth and seventh years of the child's age; though there are doubtless exceptions to this statement.

These teeth are situated in a line and just behind the deciduous teeth, and are erupted as soon as room is obtained by the elongation and expansion of the superior and inferior maxillaries. Much ignorance has prevailed, even among Dentists, and particularly in the medical profession, as to the character and importance of the preservation of these teeth. We have known instances of their extraction under circumstances unjustifiable, and unwarrantable, not to say wicked, and by those who ought to have known better.

A case in point.—Mr. P's daughter, aged eleven, was brought to our office to have the first left sup. molar extracted. At the time we were not at home. Calling upon the family physician, he extracted the tooth. A few months later, the first right sup. molar became troublesome. At once we advised the destruction of pulp and filling the cavity. To our astonishment the father did not know it to be a permanent tooth. This produced a "collision" between the physician and ourself, he insisting that these are "*shedders.*" By the aid of the books, we convinced the parent, if not the physician, that this class are permanent teeth. This is not intended to slur any member of the medical profession, unless he set himself up to know more of the dental organs, than could be expected of any student of medicine who has not made a specialty of dentistry. But to clear our way as we proceed, let it be said here, once for all, that *no* dental practitioner, extracting a first permanent

*Read at December Meeting of Mad River Valley Dental Association, 1865.

molar, without protest and a laudable effort for its preservation, is excusable, unless the parent takes all responsibility; and even then, it is heretical practice. Money ought not to induce any one to do that which is wrong, abstractly or relatively. We unhesitatingly pronounce any dental practitioner guilty of mal-practice, who, without protest, and without effort to save, extracts *any* tooth. How much more guilty is that operator, who, to gratify a whim of parent, or save himself the trouble of the "curative process," extracts a sixth year molar.

The case alluded to is not an ideal one; name, place, time, and proof, could be adduced if required. The pulp was devitalized, removed, canal and cavity thoroughly cleansed, and washed with an anti-septic, then filled. A few months ago, though three years have elapsed since filled, it was doing well.

An interesting question arises, as to the cause of the early decay of these teeth. We are not capable of an extensive or elaborate reply. The ablest pens in the profession are shedding light upon this momentous question. Doubtless poverty of the mother's milk in "earthy constituents," and the kind of food furnished the child, immediately before the eruption of these teeth, have much to do with their imperfect formation and consequent early decay, organized, as they are, advanced, and erupted immediately upon and during the nursing period. Anterior to this, however, it should have been stated, that the proverb, as old as the sacred oracles, "the fathers have eaten sour grapes and the children's teeth are set on edge," has more to do with the poverty of dentification, (as Mr. C., in the November No. of the Register, would say,) than at first might be supposed. No doubt, every operator of experience at least, has noticed the striking similarity of children's teeth to those of their parents. Can the child be expected to have inherited a good set of teeth of parents not themselves possessed of good dental organs? Of course not.

"Whatsoever a man soweth, that shall he also reap," is not less true in physical generation than in spiritual matters. But, thirdly: we don't have use enough for our teeth! What effort is required to chew our food? Do puddings, cakes, blanc-manges, pastries, floating islands, mashed potatoes, and spongy light bread, afford developing exercise to our masticating organs? Some one has said, "war is a great demonstrator." Is not exercise a great developer? For this reason the man who uses tobacco has better teeth than another whose teeth lie idle in his mouth; other things being equal, (no thanks to the tobacco, though.) Doubtless cooking is well enough, but the articles of food are not of the best kind for furnishing, in the first place, (beside what are needed as nutriment and calorifacients,) bone-making material, so that our teeth shall be provided with hard enough dentine; and then in the second place, give them exercise enough. We expect but little amendment with regard to our own teeth, or those immediately descending from us; yet it is our duty to do all we can for posterity. Might we suggest an article of food that abounds in two of the needed constituencies of good wholesome diet, we would name boiled wheat. The *whole grain*, boiled, is not only nutritious, but firm enough, even after having been well boiled, to give exercise to the molar teeth, well calculated to increase their density, and containing, at the same time, that which is vitally important to their perfect development—bone-making materials. Nor is it unpalatable.

Chemistry is doing much by analysis, in specifications, &c., and is shedding much light upon the constituent elements of, and materials indicated for, cell-forming and dentine-producing materials. ATKINSON, with the theory of calcification, and PAUL, of New Jersey, with his theory of "what are bone-making materials?" are, we are glad to believe, marching forward to a clearer understanding of the query, why teeth decay? And why are they not as good as the teeth of our ancestors? But, says some croaker, "what can we do but pull out the decayed teeth and insert artificial?" We answer, much every way, and 1st, educate! educate! Where? when? and how? 1st, In the office, there is the dentist's grand forum. 2nd, By your lady patients, teach them, and they gratuitously advertise all with whom they mingle. We do not, however, object to written or printed "intelligencing" upon the subject, provided it is not done quackishly. Let us, then, brethren, hie to make amends to coming generations, by teaching, 1st, That the mother, acting for the child, gives a healthful and sufficient amount of food. 2nd, That the child, coming now to act for himself, refuse such articles, by friendly assistance, as candies, preserves, rich cakes, &c., hurtful, and that continually; and be taught to use only those healthful articles of food, as have been indicated by wisdom and experience, as best calculated to build up a healthful and useful organism.

